# Airway delivery of interferon‐*γ* overexpressing macrophages confers resistance to *Mycobacterium avium* infection in SCID mice

**DOI:** 10.14814/phy2.13008

**Published:** 2016-11-17

**Authors:** Rajamouli Pasula, Bradley E. Britigan, Banurekha Kesavalu, Maher Y. Abdalla, William J. Martin

**Affiliations:** ^1^ Department of Internal Medicine University of Cincinnati College of Medicine Cincinnati Ohio; ^2^ Research Service VA Medical Center – Nebraska/Western Iowa Omaha Nebraska; ^3^ Department of Internal Medicine and Microbiology and Immunology University of Nebraska Medical Center Omaha Nebraska; ^4^ Department of Pathology and Microbiology College of Medicine University of Nebraska Medical Center Omaha Nebraska; ^5^ College of Public Health The Ohio State University Columbus Ohio

**Keywords:** Alveolar macrophages, immunosuppression, interferon gamma, *M. avium*, reconstitution, ventilation

## Abstract

*Mycobacterium avium* (*M. avium*) causes significant pulmonary infection, especially in immunocompromised hosts. Alveolar macrophages (AMs) represent the first line of host defense against infection in the lung. Interferon gamma (IFN‐*γ*) activation of AMs enhances in vitro killing of pathogens such as *M. avium*. We hypothesized that airway delivery of AMs into the lungs of immunodeficient mice infected with *M. avium* will inhibit *M. avium* growth in the lung and that this macrophage function is in part IFN‐*γ* dependent. In this study, normal BALB/c and BALB/c SCID mice received *M. avium* intratracheally while on mechanical ventilation. After 30 days, *M. avium* numbers increased in a concentration‐dependent manner in SCID mice compared with normal BALB/c mice. Airway delivery of IFN‐*γ*‐activated BALB/c AMs or J774A.1 macrophages overexpressing IFN‐*γ* into the lungs of SCID mice resulted in a significant decrease in *M. avium* growth (*P* < 0.01, both comparisons) and limited dissemination to other organs. In addition, airway delivery of IFN‐*γ* activated AMs and macrophages overexpressing IFN‐*γ* increased the levels of IFN‐*γ* and TNF‐*α* in SCID mice. A similar protective effect against *M. avium* infection using J774A.1 macrophages overexpressing IFN‐*γ* was observed in IFN‐*γ* knockout mice. These data suggest that administration of IFN‐*γ* activated AMs or macrophages overexpressing IFN‐*γ* may partially restore local alveolar host defense against infections like *M. avium*, even in the presence of ongoing systemic immunosuppression.

## Introduction

*M. avium* causes pulmonary infections in patients with underlying lung disease and in a group of older women without apparent lung disease (Field and Cowie 2006; Glassroth 2008; Kim et al. 2008; Kikuchi et al. 2009; van Duin et al. 2010). It can also cause disseminated disease in immunocompromised hosts, such as those with AIDS (Horsburgh et al. 2001; Griffith et al. 2007; Johnson and Odell 2014; Orme and Ordway 2014). The portal of entry of *M. avium* in humans is usually the lung or gastrointestinal tract (Damsker and Bottone 1985; Bartralot et al. 2000; Field and Cowie 2003). *M. avium* infections are often difficult to manage due to its capability of surviving within host macrophages.

Alveolar macrophages (AMs) play a key role in pulmonary host defense against mycobacteria (Leemans et al. 2001). AMs phagocytize and kill mycobacteria by several mechanisms, including generation of reactive oxygen intermediates (ROIs) and reactive nitrogen (RNIs) intermediates (Sato et al. 1998; Murray and Nathan 1999; Denis et al. 2005; Woo et al. 2009). However, bacterial killing of *M. avium* is variably successful (Schlesinger et al. 1990; Fenton and Vermeulen 1996; Labro 2000; Chan et al. 2001; Pieters 2001; Flynn and Chan 2003; Karakousis et al. 2004) due to the ability of *M. avium* to limit macrophage production of these chemical species and/or the organism's resistance to their cytotoxic effects (Appelberg and Orme 1993; Li et al. 2010; Miwa et al. 2014; Motamedi et al. 2014). Consequently, some organisms survive within the AM (Sangari et al. 1999).

Cell‐mediated immunity, characterized by activation of Th1 subsets of CD4^+^ T‐cells with an increase in TNF‐*α* and IFN‐*γ* is also important in host defense against *M avium* (Jouanguy et al. 1999; Ehrt et al. 2001; Manca et al. 2001; Nau et al. 2002). IFN‐*γ*‐activated macrophages have enhanced ability to kill mycobacteria (Bonay et al. 1999; Jouanguy et al. 1999; Beltan et al. 2000; Ehrt et al. 2001; Giacomini et al. 2001; Hoal‐van Helden et al. 2001; Manca et al. 2001; Qiao et al. 2002; Karcher et al. 2008; Herbst et al. 2011). Introducing an IFN‐*γ* transgene into the lungs of SCID mice reduced their susceptibility to BCG infection (Xing et al. 2001). IFN‐*γ*‐deficient mice show increased susceptibility to mycobacterial infection (Flynn et al. 1993; Kamijo et al. 1993; Newport et al. 1996; Sugawara et al. 1998). Humans gene mutations that disrupt IFN‐*γ* signaling enhances susceptibility to *M. avium* (Haverkamp et al. 2006). TNF‐*α* is another key factor in controlling mycobacterial infection (Chen et al. 1992; Kasuga‐aokii et al. 1999). Humans receiving anti‐TNF therapies for rheumatoid arthritis or other diseases are at increased risk for infection with mycobacteria and other intracellular pathogens (Keane 2001). Thus, TNF‐*α* and IFN‐*γ* are important in resistance to mycobacterial infection.

Both AMs and T‐cell‐derived cytokines are necessary for optimal host defense against mycobacteria. However, how these two host defense systems interface remains unclear. Cell‐mediated immunity can be restored by reconstituting normal cells into immunodeficient mice. CD4^+^ cells reconstituted into SCID mice resulted in resolution of *P. carinii* infection (Theus et al. 1995; Keane 2001). Activated macrophages have been reconstituted into humans for cancer therapy (Eymard et al. 1996; Monnet et al. 2002; Suzuki et al. 2014). However, this approach has not been evaluated for treatment or prevention of pulmonary *M. avium* infection.

Although present, AMs are not fully functional in SCID mice and are felt to contribute to the susceptibility of these mice to interfere with *M. avium* (Armstrong and Cushion 1994). The aim of this study was to evaluate whether airway delivery of normal AMs or macrophages overexpressing IFN‐*γ* decreases susceptibility to *M. avium* lung infection in SCID mice and IFN‐*γ* knockout mice (IFN‐*γ* KO). We found that transfer of IFN‐*γ* ex vivo activated AMs or macrophages overexpressing IFN‐*γ*, but not unstimulated macrophages, into lungs of SCID or IFN‐*γ* KO mice markedly enhances the ability of these animals to control local pulmonary *M. avium* infection, as well as limit disseminated disease. This protective effect is associated with a significant increase in lung levels of IFN‐*γ* and TNF‐*α*.

## Materials and Methods

### *M. avium* cultivation and isolation

*M. avium* ATCC strain 25291 (Rockville, MD) was grown in Middlebrook 7H9 broth containing albumin (50 g/L), dextrose (20 g/L), and catalase (0.03 g/L) as enrichments (BD Diagnostics, Franklin Lakes, NJ). *M. avium* was cultured at 37°C in dispersed form and harvested in mid‐log phase. The mixture was centrifuged (3500 × *g* for 30 min) at 4°C, washed with saline, and sonicated (15 sec at 20 W, GenProbe, San Diego, CA) to disrupt bacterial clumps, as described in our previous studies (Pasula et al. 2002b). The purity of the *M. avium* cultures was verified by Kinyoun staining (Midlantic Biomedical Inc., Paulsboro, NJ). The final bacterial suspension was adjusted to contain 1 × 10^9^ organisms/mL using a Spectronic 20 as previously described (Dhople and Ibanzez 1995). The accuracy of these measurements was confirmed with a serial dilution and determination of colony‐forming units (CFUs).

### Labeling of *M. avium* with FITC

*M. avium* were labeled with fluorescein isothiocyanate (FITC) (Sigma Chemical Co. St. Louis, MO) as described (Ezekowitz et al. 1991; Weaver et al. 1996). Briefly, *M. avium* was isolated as described in the preceding section. A final concentration of 1 × 10^8^/mL *M. avium* was suspended in PBS (pH 7.2) containing FITC (0.1 mg/mL) and incubated for 4 h in the dark at 37°C with occasional agitation/shaking. After the incubation, the labeled *M. avium* was centrifuged at 3000 × *g* for 20 min. The supernatant was discarded and the pellet was washed three times with PBS to remove unincorporated FITC. The labeling was verified by the direct examination under the fluorescent microscope. The final pellet was resuspended in PBS.

### Mice

Pathogen‐free 6–8‐week‐old BALB/c, BALB/c SCID (Harlan Sprague‐Dawley, Indianapolis, IN) and IFN‐*γ* KO mice with the respective BALB/c background mice were purchased from Jackson Laboratories (Bar Harbor, ME). The mice were housed and maintained under pathogen‐free conditions. All animal procedures were approved by the University of Cincinnati Institutional Animal Care and Use Committee. The mice were housed in a barrier facility, maintained in sterile conditions in microisolator cages, and supplied with sterilized food and water. All experimental animals were maintained in microisolator cages until use.

### Isolation and labeling of AMs

Alveolar macrophages were isolated from normal BALB/c or BALB/c SCID mice by bronchoalveolar lavage (BAL), as described previously (Pasula et al. 1997, 2009). Briefly, mice were killed and a 20‐guge angiocath (Becton Dickinson, Sandy, UT) was inserted into the trachea. Following BAL with ten 1 mL aliquots, BAL cells were harvested by centrifugation, washed, and then enumerated by hemocytometer (Reichert, Buffalo, NY). AM viability was determined to be >98% using trypan blue exclusion. Cytopreparation smears were made and stained with Hema III stain (Biochemical Sciences Inc., Swedesboro, NJ) to determine cellular differentials. Only the cellular suspensions that contained >98% pure AM population were used.

In some experiments, donor AMs were labeled with DiI as previously described (Pasula et al. 2002a). In selected studies, donor AMs from BALB/c mice, were incubated in the presence or absence of IFN‐*γ* (100 U/mL) for 18 h at 37°C. The production of genetically engineered J774A.1 macrophages overexpressing IFN‐*γ* from our laboratory was described previously (Wu et al. 2001). Donor AMs or AMs labeled with DiI and or J774A.1 macrophages overexpressing IFN‐*γ* were intratracheally (I.T.) administered at a concentration of 5 × 10^5^/50 μL into the mechanically ventilated SCID mice as described below. Our previous studies demonstrated that AMs labeled with DiI retain the label fastidiously in the lung (Pasula et al. 2002a), similar to reports of others using DiI as cell marker in vivo (Honig and Hume 1986, 1989; Heredia et al. 1991; Ragnarson et al. 1992) and that DiI labeling of AMs and airway delivery of AMs does not alter the function of these AMs in vivo (Pasula et al. 2002a).

### Administration of AMs, IFN‐*γ*‐treated AMs, or J774A.1 macrophages overexpressing IFN‐*γ* into recipient mice

Alveolar macrophages were administered into the lungs of mice as described in our earlier studies (Pasula et al. 2002a). Briefly**,** mice were anesthetized with isoflurane provided by the Laboratory Animal Medical Services at the University of Cincinnati, College of Medicine, following which the trachea was exposed using blunt dissection. When the mice had reached a sufficient level of anesthesia, they were surgically prepared, and given preemptive analgesics subcutaneously (Buprenorphine). A midline neck incision was made, the trachea was exposed using blunt dissection, and a 20‐guge angiocath was inserted into the trachea. The mice experienced no discernible pain. The mice were connected to the ventilator (Analytic Specialties, St. Louis, MO) that was directly connected to the airway catheter. The ventilator is time‐cycled with pressure limits set at a maximum peak inspiratory pressure of 4 cmH_2_O, a respiratory rate of 150 breaths per minute, and an inspiratory to expiratory ratio of 1:2. This results in a tidal volume of 150–250 μL per breath. Mice were ventilated for 10 min and then allowed to recover. Mechanical ventilation insures adequate delivery and distribution of mycobacteria or macrophages to the lower respiratory tract and avoids problems associated with uneven recovery from anesthesia among the animals. The ventilator has proved very safe for the mice from lung‐related injury from manipulation and the recovery is typically very fast (Martin et al. 2005). The AMs (5 × 10^5^) with or without DiI labeling in 50 μL HBSS were administered into the lungs via the tracheal catheter. The cell suspension was followed by a 200 μL of air to clear the angiocath and upper respiratory tract of the mice and the mice were ventilated for 10 min during the administration. Selected groups of mice were administered with, IFN‐*γ* AMs or J774A.1 macrophages overexpressing IFN‐*γ* or control BALB/c AMs or J774A.1 macrophages.

### Infection of mice with *M. avium*

Specific concentrations of *M. avium* (5 × 10^5^ to 1 × 10^8^) suspended in 50 μL HBSS were I.T. administered into the lungs of the mice via a tracheal catheter (Pasula et al. 2009). The mice were ventilated for another 10 min and then allowed to recover. In experiments to determine the ability of AMs or ex vivo IFN‐*γ*‐activated AMs (100 U/mL) or macrophages overexpressing IFN‐*γ* to inhibit *M. avium* growth in SCID or IFN‐*γ* KO mice, AMs (5 × 10^5^) were I.T. administered into the lungs of BALB/c and BALB/c SCID mice or IFN‐*γ* KO mice via the angiocath in the trachea. Ten minutes after administration of AMs, mice were challenged with *M. avium* (1 × 10^6^) and connected to the ventilator, as previously described (Weaver et al. 1996; Pasula et al. 2002a). Control groups included saline alone, treated SCID/BALB/c mice, *M. avium*‐infected SCID mice, *M. avium*‐infected BALB/c mice, SCID mice AMs into native recipient SCID mice or control BALB/c mice AMs into BALB/C mice. Thirty days later, mice were killed, lungs were removed, and *M. avium* was determined from lung homogenates by CFU. The initial *M. avium* inoculum that was used to infect the mice for each experiment was verified routinely by plating onto 7H11 Middlebrook plates. To assess *M. avium* growth, the lungs, liver, spleen, and kidneys were removed aseptically at specified time points. The mice were not sick at the time of measuring *M. avium* burden from the tissues as the mice were administered with the sublethal concentrations of *M. avium*. The organs were cut into small pieces and homogenized using a Tissue Tearor (Dremel, Racine, WI). *M. avium* numbers in the organ homogenates of infected mice were measured using both conventional colony‐forming units (CFU) and the radiometric BACTEC methods (BACTEC 460 TB system, Becton Dickenson Diagnostic Instrument Systems, Sparks, MD), as described in our earlier studies (Pasula et al. 1999, 2009).

### Radiometric BACTEC assay

The BACTEC method is based on the metabolism of the^14^C substrate present in the medium by viable mycobacteria and subsequent release of ^14^CO_2_ into the atmosphere above the medium. The organ homogenates (0.5 mL) of infected mice were inoculated into BACTEC 12 Middlebrook vials (Becton Dickenson Diagnostic Instrument Systems, Sparks, MD) containing 0.1 mL PANTA, an antibiotic supplement (Becton Dickinson Diagnostic Instrument Systems) which prevents the growth of any contaminating organisms. The vials were incubated at 37°C, with growth of *M. avium* determined every 24 h by a 460 BACTEC TB instrument (Becton Dickinson Diagnostic Instrument Systems) and expressed as a growth index from 0 to 999, with an index >10 indicating significant growth. In selected experiments, we used this BACTEC method, which is more sensitive than CFU when the number of surviving organisms is low and provides more rapid detection. However, for most of the studies, we used the standard conventional agar and colony counting method to provide more accurate quantification.

### Quantitation of colony‐forming units

Bacterial growth was also assessed by measuring *M. avium* colony‐forming units (CFU) in the tissue homogenates of infected mice. Serial dilutions from the tissue homogenates were prepared and plated on 7H11 agar in triplicate. The plates were incubated at 37°C, 5% CO_2_, for 2–3 weeks. The colonies were counted manually.

### Attachment/phagocytosis of *M. avium* by AMs

The attachment/phagocytosis of *M. avium* by AMs was evaluated both in vitro and in vivo. To determine the difference in the ability of normal AMs and SCID AMs to attach/phagocytose *M. avium*, an assay of attachment/phagocytosis of *M. avium* by AMs was performed as previously described (Pasula et al. 1997, 2002b; Hartmann et al. 2001). Freshly isolated AMs from BALB/c and BALB/c SCID mice were incubated with *M. avium* at 1:10 ratio (AM:*M. avium*) for 2 h at 37°C in tissue culture glass slides (BD Falcon, Becton and Dickenson). The slides were washed to remove any unbound *M. avium*. The adherent AMs on the glass slides containing bound *M. avium* were identified by Auramine O stain (Scientific Device Laboratory, Inc. Des Plaines, IL). The dye binds to the mycolic acid in the mycobacteria cell wall, and the organisms appear as bright yellow, luminous rods against a dark background. The AMs were visualized under fluorescent microscope (Orca ER Zeiss, Thornwood, NJ) to determine the attached/phagocytosed *M. avium* by the AMs and calculated as number of bacilli per AM. Five fields of 100 AMs were counted and the mean percentage of *M. avium* attached/phagocytosed by AMs was determined.

For the in vivo assessment*,* recipient mice were killed and BAL was obtained at 1, 7, 14, or 28 days following AM administration. BAL containing both administered DiI labeled AMs and resident unlabeled AMs associated with FITC‐labeled *M. avium* organisms was analyzed using a FACScan flow cytometer (Becton Dickinson, San Jose, CA). Suspensions of unlabeled AMs, DiI‐labeled AMs, and FITC‐labeled *M. avium* organisms were independently analyzed as standards for the flow cytometry studies. The percentage of *M*. avium associated with the total cells in suspension belonging to each group was determined.

### Assay of IFN‐*γ* and TNF‐*α*

IFN‐*γ* and TNF‐*α* concentrations were measured in cell‐free supernatants of BAL samples. The IFN‐*γ* or TNF‐*α* levels were determined by using commercially available ELISA kits (R&D System, Minneapolis, MN) according to the manufacturer's instructions. Cytokine levels were expressed as pg/ml of the returned BAL. Specific mRNA transcripts for IFN‐*γ* or TNF‐*α* were also determined by PCR from the lung homogenates (IDT, Coralville, IA). Specific cytokine in RNA was also determined by PCR from the lung homogenates (IDT, Coralville, IA). The expression of IFN‐*γ* mRNA was detected by RT‐PCR (Kuroda et al. 2002). The total RNA was extracted from the lungs using RNeasy Plus Mini Kit (Qiagen, Valencia, CA). The concentrations and purities of the RNA were determined by spectrophotometer (GeneQuant II, Pharmacia Biotech, San Francisco, CA) using absorbance at 260 and 280 nm (A_260_/A_280_ ratio). cDNA was synthesized using SuperScript^®^ III First‐Strand Synthesis System (Invitrogen, CA). The resultant cDNA was subjected to PCR using the following primers.

The cDNA was generated from each RNA sample (5 μg) was reverse transcribed using 2 μL of oligo(dT) _12‐18_ primer, 1 μL (200 U) of SuperScript II reverse transcriptase, 1 μL of 5 mmol/L (each) deoxynucleoside triphosphate, 5 μL of 0.1 mol/L dithiothreitol, 10 μL of 5× enzyme buffer (Gibco BRL, Grand Island, NY), and 22 μL of DEPC‐treated water (total reaction volume, 50 μL). Negative controls with all components but without reverse transcriptase and positive controls (RNA from Gibco BRL) were used for establishing reaction conditions.

The amplification mixture for each sample was made to a total volume of 50 μL. The reaction mixture contained 0.5 μL (1 μg/μL) each of a 3′ and a 5′ gene‐specific primer. For IFN‐*γ*, the sequences used were 5′‐TGA ACG CTA CAC ACT GCA TCT TGG (sense) and CGA CTC CTT TTC CGC TTC CTG AG‐3′ (anti‐sense); TNF alpha 5′‐GAG TGA CAA GCC TGT AGC CCA TGT TGT AGC A‐3′ (sense) and GCA ATG ATC CCA AAG TAG ACC TGC CCA GAC T‐3′ (anti‐sense) *β*‐actin (used as internal control) 5′‐ ACC AAC TGG GAC GAC ATG GAG AA (sense) and GTG GTG GTG AAG CTG TAG CC ‐3′ (anti‐sense) (Integrated DNA technologies, Inc, Coralville, IA), 0.5 μL (5 U/μL) of *Taq* DNA Syber green polymerase (Gibco BRL), 2.5 μL of cDNA, 21 μL of DEPC‐treated water, and 25 μL of the corresponding premix tube of FailSafe PCR (Epicentre Technologies, Madison, WI). A control RNA was used to verify the reaction is working. PCR was performed using cDNA from AMs or GAPDH cDNA using an ABI 7700 real time system (Applied Biosystems, Foster city, CA) under the following conditions: 38 cycles of 30 sec denaturation at 94°C for 5 min, 94°C for 1 min, primer annealing at 60°C for 1 min, extension at 72°C for 1 min, and a final extension at 72°C for 10 min. The PCR products were visualized on a 1.2% agarose gel stained with ethidium bromide.

### Statistical analysis

**Figure 1:**
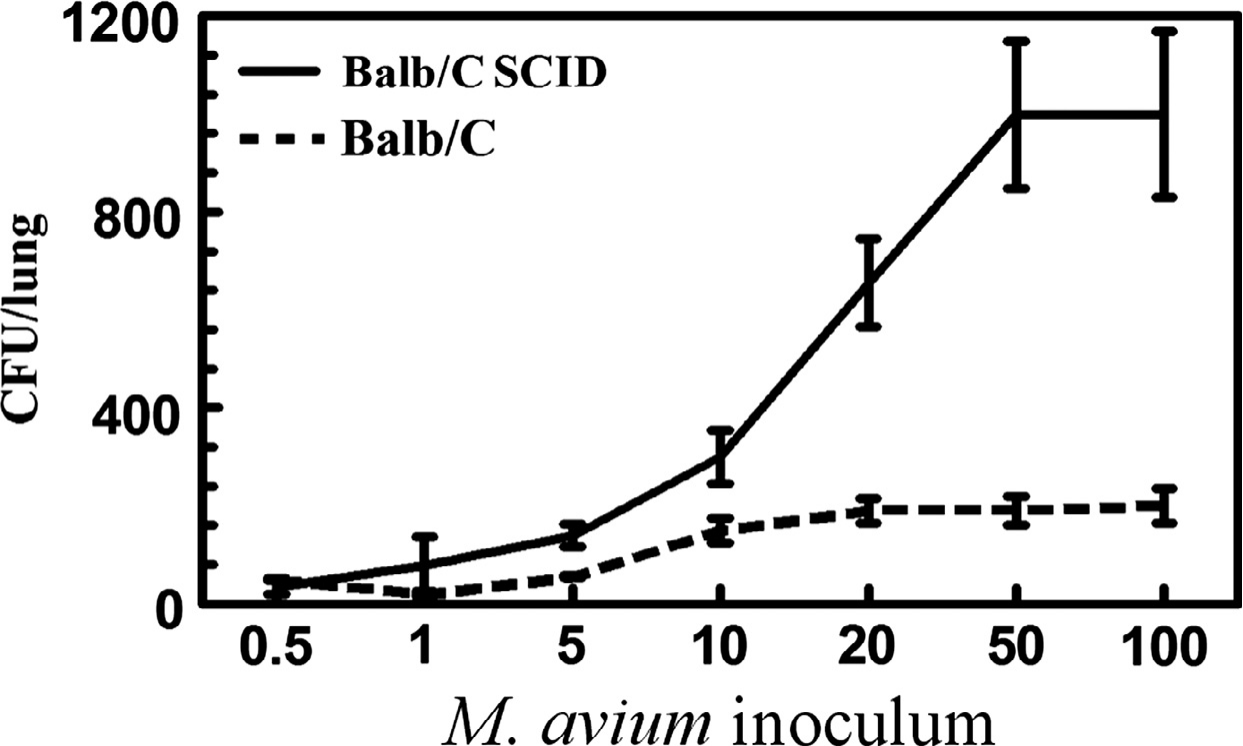
Concentration‐dependent *M. avium* infection in BALB/c and BALB/c SCID mice. To compare the susceptibility of BALB/c and BALB/c SCID mice to *M. avium* infection *M. avium* (5 × 10^5^ to 1 × 10^7^) was administered I.T. into mechanically ventilated BALB/c mice or BALB/c SCID mice. The mice were killed 30 days later and the lungs were isolated. The number of *M. avium* was determined by CFU. The results show that *M. avium* infection significantly increased in a manner directly proportional to the inoculum in BALB/c SCID mice, with maximum infection occurring at 1 × 10^7^ *M. avium*/mouse. In contrast, the extent of *M. avium* infection was lower in BALB/c mice. Results are representative of three separate experiments, and each time group contains five mice. The data are expressed as mean ± SEM. (*P* < 0.05) (determined by *T*‐test analysis).

**Figure 2:**
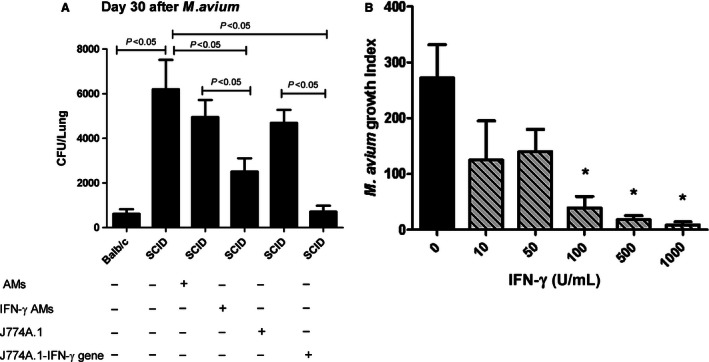
Effect of reconstitution with normal IFN‐*γ*‐activated AMs or J774A.1 macrophages overexpressing IFN‐*γ* on *M. avium* growth. (A). BALB/c *SCID* mice received I.T. 5 × 10^5^ normal IFN‐*γ*‐treated AMs (100 U/ml) from control BALB/c mice or J774A.1 macrophages genetically engineered to express IFN‐*γ*. Following instillation of AMs, mice were infected 10 min later with *M. avium* (1 × 10^6^). After 30 days, the lungs were isolated and the number of *M. avium* was determined as CFU. (A) BALB/c SCID mice infected with *M. avium* alone had more (*P* < 0.05) *M. avium* growth than normal BALB/c mice. BALB/c SCID mice that received normal AMs showed slight decrease in *M. avium* growth (*P* > 0.05). However, IFN‐*γ*‐activated AMs and macrophages overexpressing IFN‐*γ* showed a significant decrease in *M. avium* growth (*P < 0.05 and **P < 0.001 vs. SCID mice, respectively). Results are representative of three separate experiments, and each time group contains five mice and the data are expressed as mean ± SEM. (B) BALB/c SCID mice received I.T. AMs from normal BALB/c mice treated in vitro with IFN‐*γ* (0–1000 U/ml) followed 10 min later by I.T. *M. avium* (1 × 10^6^). *M. avium* growth was measured by BACTEC assay and expressed as growth index. BALB/c AMs treated with 10 U/mL of IFN‐*γ* administered to SCID mice showed a minimal (statistically not significant) decrease in *M. avium* growth. However, normal AMs activated with either 100, 500, and 1000 U/mL of IFN‐*γ* showed a significant decrease in *M. avium* growth compared with level of infection in control BALB/c SCID mice (*P* < 0.001). Results are representative of three separate experiments, and each time group contains five mice and the data are expressed as mean ± SEM. (determined by one‐way analysis of variance (ANOVA) with Bonferroni *post hoc* tests).

**Figure 3:**
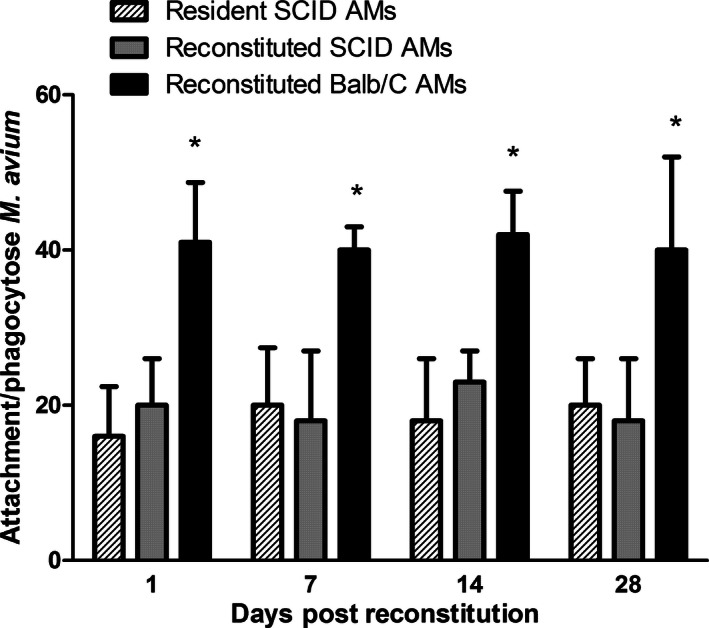
BAL evidence that reconstituted DiI‐labeled AMs attach/phagocytose *M. avium*. AMs obtained from normal BALB/c mice were labeled with DiI and instilled at a concentration of 5 × 10^5^/50 μL into the airway of mechanically ventilated BALB/c SCID mice. After 1, 7, 14, or 28 days following the reconstitution of DiI‐labeled AMs, mice were administered with FITC‐labeled *M. avium* (1 × 10^7^/50 μL) and ventilated for 10 min. The mice were killed 1 h post administration of *M. avium*. BAL was performed and attachment/phagocytosis of FITC *‐M. avium* was quantified by FACS analysis for donor DiI‐labeled AMs versus unlabeled resident SCID AMs. Normal donor AMs demonstrated significantly more attachment/phagocytosis o*f M. avium* than resident SCID AMs for each concentration of *M. avium* (*P* < 0.05). Results are representative of three separate experiments performed in triplicate. The data are expressed as mean ± SEM. (determined by one‐way analysis of variance (ANOVA) with Bonferroni *post hoc* tests).

All experiments were performed in triplicate, with each experiment repeated three times. All animal experiments were carried out using groups of at least five mice and were repeated three times. The results were expressed as means ± SEM. The statistical difference between the control and experimental data were determined with the Student's *t* test (Fig. 1) or ANOVA (Figs. 2, 3, 5, 6 and Table 1). A *P* value of less than 0.05 was considered to be statistically significant (Kuebler and Smith 1976).

**Table 1:** Effect of reconstitution of IFN‐*γ* overexpressing macrophages on dissemination of *M. avium* in BALB/c *scid* mice

SCID + *M. avium*	Spleen	Liver	Kidney
Control	*4725 ± 1088	*3775 ± 757	*4331 ± 863
J774A.1	**3825 ± 624	**3253 ± 710	**4225 ± 531
J774A.1 + IFN‐γ gene	***420 ± 153	***271 ± 50	***362.5 ± 103

*M. avium* (10^6^/50 μL) were administered by airway delivery followed by an administration of macrophages overexpressing IFN‐*γ* into the BALB/c *scid* mice. Thirty days later, *M. avium* growth was assessed by CFU from liver, kidney, and spleen homogenates. The *M. avium* growth slightly decreased in BALB/c *scid* mice alone or mice administered with control macrophages. However, *M. avium* growth was significantly decreased in BALB/c scid mice administered with macrophages overexpressing IFN‐*γ* (*P < 0.05, **P < 0.05, ***P < 0.001). The results are expressed as the mean ± SEM (*n* = 3) of three experiments performed in triplicate (determined by one‐way analysis of variance (ANOVA) with Bonferroni *post hoc* tests).

## Results

### Effect of AMs on *M. avium* infection in SCID mice

*M. avium* infection levels in the lungs of SCID mice increased significantly (*P* < 0.05) with the concentration of the initial airway‐delivered inoculum compared and at each inoculum were significantly greater (*P* < 0.05) than seen with wild‐type mice (Fig. 1). Thus, this model of *M. avium*‐infected SCID mice allowed us to test the hypothesis that normal immunocompetent AMs with or without activation by IFN‐*γ* were important in the control of *M. avium* lung infection.

To determine the impact of the presence of normal AMs in the control of *M. avium* in SCID mice, AMs from BALB/c mice were instilled into the lungs of recipient SCID mice followed 10 min later by airway delivery of *M. avium*. Four weeks later, there was a slight, but not significant, decrease in *M. avium* numbers in the lungs of the AM‐treated SCID mice relative to untreated control (Fig. 2A).

To test the hypothesis that IFN‐*γ* activation of AMs was necessary for effective host defense, AMs from normal BALB/c mice with or without treatment with IFN‐*γ* were I.T. administered into the airways of BALB/c or BALB/c SCID mice followed by airway delivery of *M. avium*. BALB/c SCID mice received I.T. AMs from normal BALB/c mice treated in vitro with IFN‐*γ* (0–1000 U/mL) followed 10 min later by I.T. *M. avium* (1 × 10^6^). BALB/c SCID mice that received AMs activated with 100, 500, and 1000 U/mL of IFN‐*γ* exhibited a significant decrease in the number of *M. avium* in their lungs at 4 weeks relative to untreated BALB/c SCID mice (*P* < 0.05) (Fig. 2B). Administration of AMs from wild‐type mice treated in vitro with 10 or 50 U/mL of IFN‐*γ* to BALB/c SCID mice exhibited a trend toward reduced growth of *M. avium*, but it was not significantly (Fig. 2B, *P* = 0.08). BALB/c SCID AMs administered to BALB/c SCID mice or BALB/c mice did not reduce *M. avium* numbers from the lungs with or without IFN‐*γ* treatment (data not shown). These results suggest that administration of IFN‐*γ*‐activated wild‐type AMs significantly enhances the ability of SCID mice to resist infection with *M. avium*.

### Effect of IFN‐*γ* overexpressing macrophages on *M. avium* infection

To test the hypothesis that macrophages actively expressing IFN‐*γ* might be effective in control of *M. avium*, J774A.1 macrophages overexpressing IFN‐*γ* were I.T. delivered into BALB/c SCID mice followed by airway delivery of *M. avium* as described above. Consistent with our earlier results, administration of normal AMs ex vivo treated with IFN‐*γ* to SCID BALB/c mice resulted in increased resistance to *M. avium* (Fig. 2A). BALB/c SCID mice receiving control J774A.1 macrophages followed by *M. avium* infection exhibited levels of *M. avium* in their lungs that was similar to untreated control animals (Fig. 2A). However, BALB/c SCID mice administered J774A.1 macrophages overexpressing IFN‐*γ* showed a significantly enhanced resistance to *M. avium* growth compared to SCID mice administered with either J774A.1 macrophages or AMs ex vivo treated with IFN‐*γ* (*P* < 0.05) (Fig. 2A). These results suggest that delivery of IFN‐*γ*‐activated normal AMs or J774A.1 macrophages overexpressing IFN‐*γ* to the airway of SCID mice enhances their ability to resist infection by *M. avium* with IFN‐*γ* overexpressing macrophages being the most efficacious.

### Administration of AMs reduces *M. avium* dissemination in SCID mice

In SCID mice, pulmonary infection with *M. avium* disseminates to other organs (e.g., liver, spleen and kidney). Therefore, *M. avium* CFU in non‐pulmonary organs were also determined in homogenates obtained from liver, kidney, and spleen 30 days post AM administration. As expected, there was significant *M. avium* growth in the liver, spleen, and kidneys of control SCID mice (Table 1). SCID mice administered normal AMs did not show any significant difference in *M. avium* in these organs relative to untreated control SCID mice. In contrast, administration of IFN‐*γ*‐stimulated AMs to the SCID mice with significantly inhibited the *M. avium* growth (data not shown) and this growth was further decreased in mice administered J774.1macrophages overexpressing IFN‐*γ* (Table 1). These data suggest that macrophages overexpressing IFN‐*γ* enhanced alveolar host defense during immunosuppression and limited the dissemination of *M. avium* infection.

### Attachment/phagocytosis of *M. avium* by reconstituted AMs

To determine the functionality of the AMs possible mechanisms by which adoptive transfer of AMs from normal mice to SCID mice decreases *M. avium* growth, we first compared the extent to which *M. avium* are phagocytized by normal AMs and SCID AMs. AMs were isolated from BALB/c and BALB/c *SCID* mice and then incubated with *M. avium*. After 2 h, significantly more *M. avium* were attached/phagocytosed by the BALB/c AMs (197 ± 28.6 bacilli/100 AMs) compared to AMs from BALB/c SCID mice (74 ± 9.2 bacilli/100 AMs, *P* < 0.05). These results suggest that normal BALB/c AMs exhibit greater ability for attachment/phagocytosis of the *M. avium* than the resident SCID AMs (*P *<* *0.05).

We next determined if this enhanced ability of normal AMs to phagocytize *M. avium* in vitro could be demonstrated in vivo in the lungs of SCID mice. To differentiate normal AMs instilled into the airway of SCID mice from endogenous SCID AMs, normal AMs were Dil‐labeled prior to transfer to the SCID mouse airway. As shown in Figure 3, DiI‐labeled AMs from normal BALB/c mice demonstrated significantly better attachment/phagocytosis of FITC‐labeled *M. avium* in SCID mice than the resident SCID AMs (*P* < 0.05, all comparisons). There was no difference between donor SCID AMs and resident SCID AMs in the ability to attach and phagocytose *M. avium* in SCID mice (Fig. 3) or between donor BALB/c AMs and resident AMs in BALB/c mice. A time course of attachment/phagocytosis of *M. avium* in vivo at 1, 7, 14, and 28 days post infection with *M. avium* demonstrated that donor BALB/c AMs delivered to SCID mice had significantly better attachment/phagocytosis of *M. avium* compared to resident BALB/c SCID AMs at each time point (Fig. 3).

**Figure 4:**
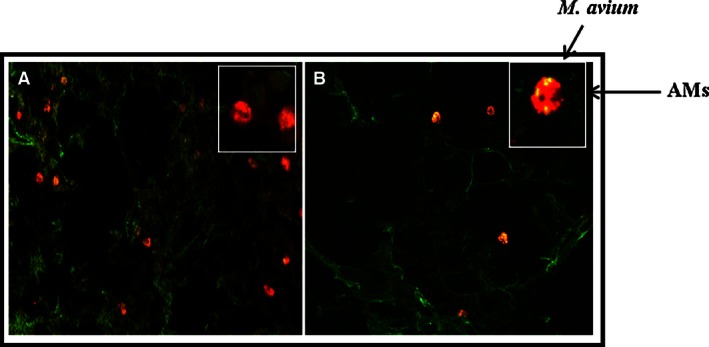
Morphological evidence that DiI‐labeled AMs reconstituted into SCID mice attach/phagocytose *M. avium*. BALB/c SCID mice received 5 × 10^5^ DiI labeled normal BALB/c AMs or BALB/c SCID AMs and 24 h later FITC‐labeled *M. avium* (2 × 10^7^). One hour later, the animals were killed, lungs were perfused, and the lung sections were examined by confocal microscopy. In Figure 4A, the DiI labeled donor SCID AMs and free unattached FITC‐labeled *M. avium* are visible. SCID AMs do not reveal internalization of *M. avium* (insert). In 4B, the donor normal BALB/c AMs reveal more phagocytosed *M. avium* (insert) as indicated by arrows.

Morphologic assessment of the lungs from the SCID mice demonstrated that normal BALB/c AMs effectively phagocytized FITC‐*M. avium* compared to either donor SCID AMs or resident SCID AMs (*P* < 0.05) (Fig. 4). These data provide further evidence that normal AMs delivered into the lungs of SCID mice can effectively function better and viable in the immunodeficient lung environment within the time constraints of the experiment.

### Effect of airway delivery of AMs on IFN‐*γ* and TNF levels

**Figure 5:**
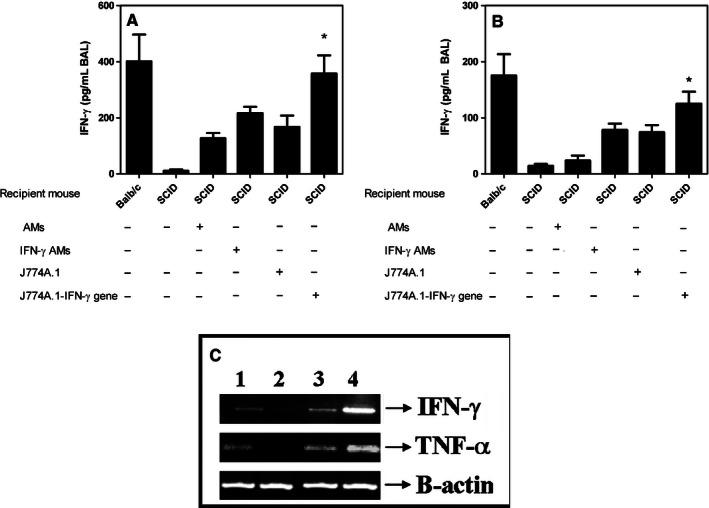
Airway delivery of normal AMs or IFN‐*γ*‐activated AMs increases IFN‐*γ* and TNF‐*α* in BAL of SCID mice. IFN‐*γ* and TNF‐*α* levels in BAL were determined 30 days following AM reconstitution and airway delivery of *M. avium*. (A). Control SCID mice had low levels of IFN‐*γ* compared to BALB/c SCID mice administered normal AMs, IFN‐*γ*‐activated AMs, J774A.1 macrophages, or J774A.1 macrophages overexpressing IFN‐*γ* (*P* < 0.05 for all comparisons vs. IFN‐*γ* levels in SCID mice). (B). There was no change in the TNF‐*α* levels in BALB/c SCID mice with or without *M. avium* challenge. The TNF‐*α* levels significantly increased in BALB/c SCID mice administered with normal AMs or IFN‐*γ*‐activated AMs or J774A.1 macrophages or macrophages overexpressing IFN‐*γ* (*P* < 0.05 for all comparisons vs. TNF‐*α* levels in SCID mice). (C). Airway delivery of macrophages overexpressing IFN‐*γ* increases IFN‐*γ* and TNF‐*α* mRNA levels in the lungs of SCID mice. IFN‐*γ* or TNF‐*α* mRNA expression was detected by PCR from lungs following macrophage reconstitution and challenge with *M. avium*. Shown are the results for SCID mice that: received *M. avium* (Lane 1); received J774A.1 cells but were not challenged with *M. avium* (Lane 2); received J774A.1 macrophages overexpressing IFN‐*γ*, but not *M. avium* (Lane 3); and J774A.1 macrophages overexpressing IFN‐*γ* plus *M. avium* (Lane 4). Results are representative of three separate experiments, and each time group contains five mice and the data is expressed as mean ± SEM. (determined by one‐way analysis of variance (ANOVA) with Bonferroni *post hoc* tests).

IFN‐*γ* and TNF‐*α* play an important role in host defense against mycobacterial infection. We analyzed IFN‐*γ* and TNF‐*α* levels in the lungs of SCID mice and control BALB/c mice following instillation of *M. avium* and/or AMs. IFN‐*γ* levels were significantly increased in wild‐type BALB/c mice 4 weeks following *M. avium* challenge. In contrast, SCID mice had very low levels of IFN‐*γ* before *M. avium* and this did not increase substantially with *M. avium* infection (Fig. 5A). IFN‐*γ* levels in SCID mice that had received normal BALB/c AMs or IFN‐*γ*‐activated AMs, were significantly higher at day 30 (*P* < 0.05, all comparisons, Fig. 5A). However, mice receiving either IFN‐*γ* primed AMs or IFN‐*γ* transgene expressing AMs exhibited significantly higher levels of IFN‐*γ* relative to those administered normal AMs (*P* < 0.05 both comparisons). As expected, SCID mice receiving SCID AMs had low IFN‐*γ* levels (Fig. 5A).

We next examined TNF‐*α* levels from the BALs to determine if the increase in IFN‐*γ* levels correlated with TNF‐*α* levels. SCID mice had low TNF‐*α* levels in the BAL with or without *M. avium* infection compared to control BALB/c mice (Fig. 5B). After receiving normal AMs, SCID mice demonstrated an increase in TNF‐*α* levels on day 30 (Fig. 5B) and these TNF‐*α* levels were increased to even greater levels in SCID mice that received IFN‐*γ*–activated AMs (*P* < 0.05) (Fig. 5B). SCID mice that received *M. avium* alone or SCID mice reconstituted with SCID AMs maintained low TNF‐*α* levels at all time points [data not shown].

IFN‐*γ* or TNF‐*α* mRNA expression was measured by semiquantitative PCR from murine lungs that were obtained 30 days following macrophage reconstitution and challenged with *M. avium* infection. SCID mice reconstituted with J774A.1 cells with and without *M. avium* had low detectable IFN‐*γ* or TNF‐*α* expression. SCID mice reconstituted with J774A.1 macrophages overexpressing IFN‐*γ* exhibited significantly increased IFN‐*γ* and TNF‐*α* expression (Fig. 5C). These levels were further increased in response to *M. avium* infection (Fig. 5C).

There was a direct correlation between TNF‐*α* and IFN‐*γ* levels on day 1, 7, 14, and 30 (*r* ^2^ = 0.8). Furthermore, the lung burden of *M. aviu*m in SCID mice was inversely correlated with the levels of TNF‐*α* and IFN‐*γ* (*r* ^2^ = 0.65, *P* value < 0.05). Thus, the increase in IFN‐*γ* and TNF‐*α* levels in the lungs of SCID mice reconstituted with activated AMs inversely correlated with the levels of *M. avium* organisms, suggesting that these cytokines are involved in the conferred resistance to *M. avium* infection in SCID mice.

### Effect of AMs on *M. avium* infection in IFN‐*γ* KO mice

**Figure 6:**
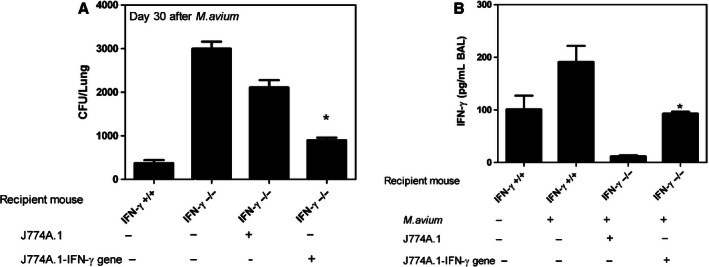
Effect of reconstitution of normal AMs or IFN‐*γ*–activated AMs in IFN‐*γ* KO mice. IFN‐*γ* KO mice underwent I.T. administration of either normal or IFN‐*γ*–activated AMs (5 × 10^5^). After 10 min, *M. avium* (1 × 10^6^/50 μL) was I.T. administered into the recipient mice. The mice were killed on day 30, following which *M. avium* CFU, as well as IFN‐*γ* and TNF‐*α* levels were determined. (A). *M. avium* CFU in the lung homogenate. There was no change in the *M. avium* growth in IFN‐*γ* KO mice. The *M. avium* growth was significantly reduced in IFN‐*γ* KO mice administered with macrophages overexpressing IFN‐*γ*. Panels (A) and (B) indicate IFN‐*γ* and TNF‐*α* levels, respectively, in BAL. There was no change in the TNF‐*α* levels in mice administered normal AMs (data not shown). IFN‐*γ* and TNF‐*α* levels were significantly increased in IFN‐*γ* KO mice administered with macrophages overexpressing IFN‐*γ* on day 30. The results are expressed as the mean ± SEM (*n* = 3) of three experiments performed in triplicate (determined by one‐way analysis of variance (ANOVA) with Bonferroni *post hoc* tests).

IFN‐*γ* KO mice and individuals with a defect in IFN‐*γ* signaling also exhibit enhanced susceptibility to mycobacterial infection (Borges et al. 2012). Given the above results, we determined whether the transfer of normal or IFN‐*γ*‐activated macrophages altered the susceptibility of IFN‐*γ* KO mice to *M. avium*. We administered normal or IFN‐*γ*‐activated AMs to IFN‐*γ* KO mice and subsequently challenged the recipient mice with *M. avium*. IFN‐*γ* KO mice and the background control mice that received normal AMs did not show any significant change in their susceptibility to *M. avium* growth as measured by determining lung CFU at 30 days post AM administration (data not shown). In contrast, IFN‐*γ* KO mice administered IFN‐*γ* overexpressing J774.1 cells had significantly fewer lung *M. avium* than those that were untreated (Fig. 6). These results show that the IFN‐*γ* KO mice are highly susceptible to pulmonary *M. avium* infection and that administration of IFN‐*γ* overexpressing macrophages diminishes *M. avium* susceptibility in these mice.

We also examined BAL samples from the same mice for the presence of IFN‐*γ* and TNF‐*α*. As expected, IFN‐*γ* KO mice had no detectable IFN‐*γ* in the BAL with or without challenge with *M. avium,* nor did IFN‐*γ* KO mice that received *M. avium* alone or IFN‐*γ* KO mice reconstituted with AMs from IFN‐*γ* KO mice (data not shown). In contrast, IFN‐*γ* KO mice that received macrophages overexpressing IFN‐*γ*, demonstrated a significant increase in IFN‐*γ* and TNF‐*α* levels [Fig. 6B and C (*P* < 0.001)]. These data suggest that IFN‐*γ* is one of the critical mediators in clearance of *M. avium* and that reconstitution of macrophages overexpressing IFN‐*γ* effectively restores the level of this critical cytokine in the IFN‐*γ* KO mice, as well as that of TNF‐*α*.

## Discussion

*M. avium* is an important opportunistic pathogen. It leads to progressive chronic pulmonary infection in patients with underlying lung disease, as well as in a subpopulation of older women (Field and Cowie 2006; Kim et al. 2008; Kikuchi et al. 2009). In immunocompromised hosts, such as those with advanced HIV infection, *M. avium* causes severe disseminated disease (Horsburgh et al. 2001; Griffith et al. 2007; Orme and Ordway 2014). Susceptibility to *M. avium* infection has been linked to impaired AM function (Bermudez et al. 1994; Nepal et al. 2006). In this study, we demonstrated that transfer of IFN‐*γ*‐activated AMs or an IFN‐*γ* overexpressing murine macrophage cell line into the lungs of two different types of immunosuppressed mice conferred enhanced resistance to lung infection and subsequent dissemination of *M. avium* following acquisition of the organism via a pulmonary route.

We have previously shown that AMs from normal mice can be transferred to the airways of SCID mice and that these reconstituted AMs exhibit enhanced phagocytic activity compared with resident SCID AMs for up to 28 days following airway delivery (Wu et al. 2001; Pasula et al. 2002a). Therefore, we assessed whether the delivery of AMs from immunocompetent mice to the airways of SCID mice would enhance resistance of these animals to *M. avium* growth in vivo despite the presence of persistent systemic immunosuppression. We found that SCID mice alone and SCID mice reconstituted with wild‐type AMs or SCID AMs became heavily infected with *M. avium*, suggesting that the simple transfer of normal AMs to SCID mice was ineffective in enhancing their resistance to the *M. avium* infection.

Optimal host response against many invading respiratory pathogens is known to depend on T‐cells and on the production of IFN‐*γ*. IFN‐*γ* is important in the control of *M. avium* infection and may act in part by enhancing the antimicrobial activity of local macrophage populations (Rose et al. 1991; Jouanguy et al. 1999; Ehrt et al. 2001). IFN‐*γ*‐deficient mice are more susceptible to *M. avium* infection (Cooper et al. 1993; Flynn et al. 1993; Kamijo et al. 1993; Newport et al. 1996), as are humans with defects in IFN‐*γ*‐mediated signal transduction. In this study, airway delivery of AMs exogenously treated with IFN‐*γ* or the use of J774A.1 murine macrophages genetically modified to overexpress IFN‐*γ* conferred significant resistance to *M. avium* infection in SCID mice and IFN‐*γ* KO mice. This effect was not limited to the *M. avium* burden in the lung, but these therapies also decreased the number of organisms recovered from distant tissue sites. Interestingly, instillation of fibroblasts constitutively expressing IFN‐*γ* into the peritoneal cavity of BALB/c mice has been previously reported to enhance resistance to pulmonary infection with *M. avium* (Kim et al. 2000). Aerosol delivery of IFN‐*γ* to human patients has also been used effectively in the treatment of pulmonary *M. avium* infection (Hallstrand et al. 2004). Our results suggest that immunocompetent AMs can partially restore alveolar host defense against *M. avium* by a process that is associated with the production of IFN‐*γ*.

TNF‐*α* production is also closely linked to resistance to mycobacterial infections (Flynn et al. 1995). In fact, the ability of IFN‐*γ* to enhance resistance to mycobacterial infection may in part be mediated by an increase in macrophage production of TNF‐*α* (Appelberg et al. 1994). The critical importance of TNF‐*α* in control of mycobacteria is underscored by the reactivation of dormant mycobacterial infection with use of TNF‐*α* monoclonal antibodies in human subjects with autoimmune diseases (Keane 2001). In this study, airway delivery of AMs activated with IFN‐*γ* or J774A.1 macrophage overexpressing IFN‐*γ* into the lungs of SCID mice or IFN‐*γ* KO mice not only increased levels of IFN‐*γ*, but also resulted in a corresponding increase in TNF‐*α* in the lungs of these mice. Furthermore, the lung burden of *M. aviu*m in these two groups of mice inversely correlated with both the levels of TNF‐*α* and IFN‐*γ*. These results support an important role for IFN‐*γ* and TNF‐*α* in control of *M. avium* infection and restoration of IFN‐*γ* and TNF production by administered AMs markedly enhances the effector function of AMs in response to *M. avium*. In this model of AM reconstitution, TNF‐*α* release by IFN‐*γ*‐activated AMs is the key to an effective AM‐mediated response to mycobacteria in vivo. We do not know if TNF‐*α* production is released from the administered AMs or due to effect of IFN‐*γ* on resident AMs or both. Further the protective effect may be the result of activation in T‐cells or other resident cells. In future studies, the use of TNF‐*α* −/− mice or use of TNF‐*α* antibodies in vivo to inhibit TNF‐*α* activity will permit direct in vivo assessment if TNF‐*α* is a critical mediator in the AM response to mycobacterial infection. Similar to this study, use of reconstituted TNF‐*α* +/+ AMs into TNF‐*α*‐deficient mice may allow us to confirm or refute this hypothesis in vivo.

In summary, we have shown that airway administration of IFN‐*γ*‐activated AMs or J774A.1 macrophages overexpressing IFN‐*γ* to SCID mice markedly enhances resistance to infection with *M. avium*. Furthermore, the inability of IFN‐*γ* KO mice to control the *M. avium* infection could also be rectified by the administration of macrophages overexpressing IFN‐*γ*. Our studies may provide a basis for developing a complementary and alternative approach to the prevention of *M. avium* infection in individuals with local or systemic immunodeficiency. Our results cannot yet be extrapolated to the efficacy of this approach for the treatment of active *M. avium* infection. Future studies are planned to determine the effect of administration of IFN‐*γ*‐producing AM in mice with established *M. avium* infection.

## Conflict of Interest

None declared.
